# Glembatumumab vedotin for patients with metastatic, gpNMB overexpressing, triple-negative breast cancer (“METRIC”): a randomized multicenter study

**DOI:** 10.1038/s41523-021-00244-6

**Published:** 2021-05-20

**Authors:** Linda T. Vahdat, Peter Schmid, Andres Forero-Torres, Kimberly Blackwell, Melinda L. Telli, Michelle Melisko, Volker Möbus, Javier Cortes, Alberto J. Montero, Cynthia Ma, Rita Nanda, Gail S. Wright, Yi He, Thomas Hawthorne, Rebecca G. Bagley, Abdel-Baset Halim, Christopher D. Turner, Denise A. Yardley

**Affiliations:** 1 Weill Cornell Medicine, New York, NY USA; 2 Center for Experimental Cancer Medicine, Barts Cancer Institute, London, UK; 3 University of Alabama School of Medicine, Birmingham, AL USA; 4 Duke University Medical Center, Durham, NC USA; 5 Stanford University School of Medicine, Stanford, CA USA; 6 University of California, San Francisco Helen Diller Family Comprehensive Cancer Center, San Francisco, CA USA; 7 Klinikum Frankfurt Hoechst, Frankfurt, Germany; 8 IOB Institute of Oncology, Quironsalud Group, Madrid & Barcelona, Vall d´Hebron Institute of Oncology (VHIO), Barcelona, Spain; 9 Cleveland Clinic, Cleveland, OH USA; 10 Washington University, St. Louis, MO USA; 11 University of Chicago, Chicago, IL USA; 12 Florida Cancer Specialists, New Port Richey, FL USA; 13 Celldex Therapeutics, Inc., Hampton, NJ USA; 14 Sarah Cannon Research Institute/Tennessee Oncology, PLLC, Nashville, TN USA; 15 Present Address: AstraZeneca, Gaithersburg, MD USA; 16 Present Address: Syndax, Waltham, MA USA; 17 Present Address: Taiho Oncology, Princeton, NJ USA; 18 Present Address: Blueprint Medicines, Inc., Cambridge, MA USA

**Keywords:** Cancer, Breast cancer

## Abstract

The METRIC study (NCT#0199733) explored a novel antibody–drug conjugate, glembatumumab vedotin (GV), targeting gpNMB that is overexpressed in ~40% of patients with triple-negative breast cancer (TNBC) and associated with poor prognosis. The study was a randomized, open-label, phase 2b study that evaluated progression-free survival (PFS) of GV compared with capecitabine in gpNMB-overexpressing TNBC. Patients who had previously received anthracycline and taxane-based therapy were randomized 2:1 to receive, GV (1.88 mg/kg IV q21 days) or capecitabine (2500 mg/m^2^ PO daily d1–14 q21 days). The primary endpoint was RECIST 1.1 PFS per independent, blinded central review. In all, 327 patients were randomized to GV (213 treated) or capecitabine (92 treated). Median PFS was 2.9 months for GV vs. 2.8 months for capecitabine. The most common grade ≥3 toxicities for GV were neutropenia, rash, and leukopenia, and for capecitabine were fatigue, diarrhea, and palmar-plantar erythrodysesthesia. The study did not meet the primary endpoint of improved PFS over capecitabine or demonstrate a relative risk/benefit improvement over capecitabine.

## Introduction

It is estimated that 268,600 new cases of breast cancer and 41,760 deaths would occur in 2019 in the United States^1^; globally, these numbers reach 2,088,849 new cases and 626,679 deaths^2^. Triple-negative breast cancer (TNBC), which under-expresses the estrogen and progesterone hormone receptors (ER, PR) and the human epidermal growth factor receptor (HER2), accounts for 15–20% of all breast cancers. Effective treatment for TNBC is limited as hormonal therapies and HER2-targeted agents are not applicable, and no approved molecularly targeted drugs were available until the approval of olaparib and talazoparib in 2018, limited to patients with a germline BRCA mutation^3,4^, or atezolizumab, targeting tumors that express programmed death (PD)-L1-positive immune cells, and the recently approved sacituzumab govitecan-hizy. Capecitabine is a standard of care option for patients with TNBC resistant to anthracyclines and paclitaxel, however, the benefit is modest with progression-free survival (PFS) reported to be 1.7–2.7 months^5–8^.

gpNMB (glycoprotein non-metastatic B) is an internalizable transmembrane protein overexpressed in ~40% TNBC and associated with a poor prognosis^9^. Preclinical studies have implicated gpNMB in tumor cell invasion, metastasis, and angiogenesis^10–12^. Glembatumumab vedotin (GV) is an antibody–drug conjugate (ADC) consisting of a fully-human gpNMB-specific IgG_2_ antibody coupled to the microtubule inhibitor monomethyl auristatin E (MMAE) via a protease-sensitive valine-citrulline peptide linker, designed to induce cell cycle arrest and cell death by releasing MMAE after internalization into the lysosomal compartment of gpNMB-expressing cells. ADCs are well-established and highly active therapeutics in breast cancer and other malignant diseases^8,13–15^.

The initial safety evaluation and recommended phase 2 dose of GV were determined in a phase 1/2 study in heavily pre-treated, advanced breast cancer (NCT# 00704158)^16^. In the subsequent phase 2 “EMERGE” study (NCT# 01156753), GV demonstrated preliminary evidence of activity in a similar patient population^17^. An exploratory biomarker analysis from EMERGE also suggested that patients with advanced TNBC overexpressing gpNMB (i.e., ≥25% tumor epithelial cells staining positive by immunohistochemistry (IHC)) were most likely to derive greater benefit from GV, compared with standard chemotherapies. Because of the exploratory nature of the analysis and the limited sample size of the cohort, the METRIC study was conducted to assess in a randomized, controlled fashion whether GV would improve PFS compared with capecitabine for patients with gpNMB-overexpressing, metastatic TNBC.

## Results

### Population and baseline characteristics

**Fig. 1:**
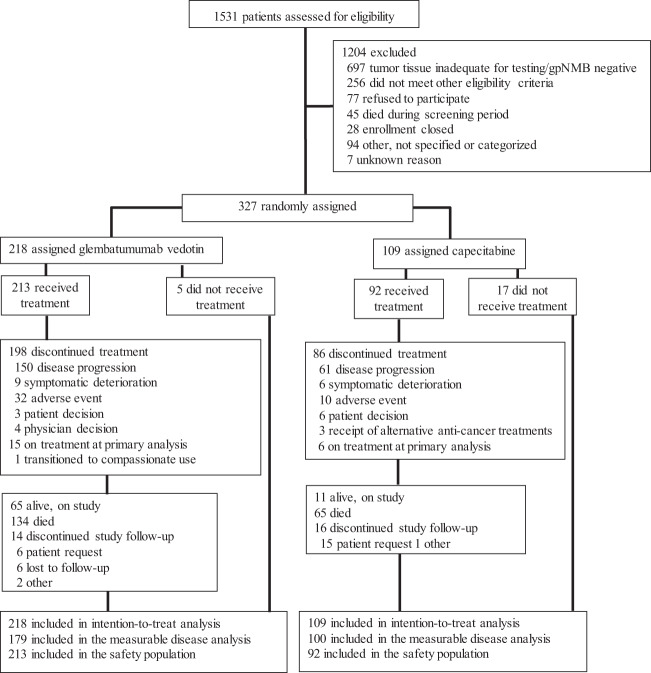
METRIC trial patient disposition.

A total of 1531 patients were screened for study eligibility (Fig. 1). Of the 1172 (77%) patients for whom tumor tissue was provided and adequate for gpNMB expression testing, 650 (55%) had tumors overexpressing gpNMB (i.e., ≥25% cells positive). Between 19 February 2014 and 21 August 2017, 327 patients were randomized to receive GV (218) or capecitabine (109) and constituted the intention-to-treat population. All patients submitted tissue collected in the advanced disease setting. In all, 109 (33%) of randomized patients had recent tumor biopsies for eligibility testing while the remaining 218 (67%) patients submitted archival tissue. Twenty-two patients, 5 in the GV arm and 17 in the capecitabine arm, were randomized but did not receive study treatment, owing to concern over randomization to the control arm and failure to meet eligibility. Thus, 213 and 92 patients in the GV and capecitabine arms, respectively, were included in the safety population. Although eligibility for enrollment (including confirmation of measurable baseline disease) was determined by the center, the imaging review committee (IRC) assessment determined the measurable disease population. A total of 179 patients in the GV arm and 100 patients in the capecitabine arm were assessed by the IRC to have at least one RECIST 1.1 measurable lesion at baseline and constituted the measurable disease population.

Based on the accumulation of events, 30 November 2017 was pre-selected as the cutoff for the primary analysis. After all data were read and cleaned through this date, a total of 223 PFS events had accumulated and were utilized for data analysis.

Pretreatment demographic, baseline, and disease characteristics were well balanced between treatment groups (Tables 1 and 2), with exception of the determination of patients with measurable disease by IRC, as noted above. All enrolled patients were female with a median age of 55 years. At study entry, most patients (77%) had visceral disease and 50% had ≥3 sites of disease. All but one patient had received taxane and 86% had prior anthracycline. Patients had received a median of 1 (range 0–5) prior anticancer therapies for advanced disease. Sixty percent of patients progressed within 6 months from the end of their last cytotoxic-containing regimen with 40% of patients having the best response of the progressive disease.

**Table 1:** Pretreatment patient characteristics.

	Glembatumumab vedotin (*n* = 218)		Capecitabine (*n* = 109)		All patients (*n* = 327)	
	No.	%	No.	%	No.	%
*Age, years*						
Median	55		55		55	
Range	28–85		31–85		28–85	
*ECOG performance status*						
0	106	49	60	55	166	51
1	111	51	47	43	158	48
2	1	1	1	1	2	1
Unknown	0	0	1	1	1	<1
Visceral disease^a^	173	79	80	73	253	77
*Duration of disease since initial diagnosis of breast cancer, years*						
Median	2.5		2.3		2.4	
Range^b^	0.3–30.9		0.3–35.3		0.3–35.3	
*Duration of metastatic disease, years*						
Median	0.6		0.6		0.6	
Range	0–8.0		0–3.4		0–8.0	
Historic or current CNS involvement	20	9	6	6	26	8
R*eceptor status for metastatic disease*^c^						
Triple negative (ER, PR, HER2)	216	99	108	99	324	99
ER/PR < 1%	189	87	98	90	287	88
ER/PR 1–9%	28	13	11	10	39	12
*gpNMB expression by IHC*^d^						
<25%			1	1	1	<1
25–49%	78	36	43	39	121	37
50–<75%	69	32	29	27	98	30
75–100%	71	33	36	33	107	33
*No. of prior anticancer regimens*^e^						
Median	2		2		2	
Range	1–5		1–5		1–5	

*ECOG* Eastern Cooperative Oncology Group, *ER* estrogen receptor, *PR* progesterone receptor, *HER2* human epidermal growth factor receptor 2, *gpNMB* glycoprotein NMB.

^a^Visceral disease: tumor in lung, liver, spleen, esophagus, stomach, small intestine, colon, rectum, omentum, peritoneum, kidney, pancreas, or adrenal gland.

^b^Time from initial diagnosis of breast cancer, including patients who were not triple-negative at the time or whose receptor status was unknown.

^c^Triple-negative status was unknown for two patients in the glembatumumab vedotin arm and one patient in the capecitabine arm. ER/PR % positivity was unknown for one patient in the glembatumumab vedotin arm.

^d^gpNMB: based on % expression in malignant epithelial cells from the last sample before first dose of study drug. The highest expression is reported there was ≥1 sample collected with the same date.

^e^Including hormonal therapies.

**Table 2:** Prior treatments.

	Glembatumumab vedotin (*n* = 218)		Capecitabine (*n* = 109)		All patients (*n* = 327)	
	No.	%	No.	%	No.	%
*No. of prior cytotoxic-containing regimens in metastatic setting*						
Median	1		1		1	
Range^a^	0–3		0–4		0–4	
*No. of prior relapses in the advanced setting*						
0	45	21	21	19	66	20
1	122	56	58	53	180	55
2	42	19	24	22	66	20
3	9	4	6	6	15	5
Prior anthracycline	185	85	95	87	280	86
Prior taxane						
*No. of prior taxane therapies*						
0	0	0	1	1	1	<1
1	164	75	74	68	238	73
2	51	23	33	30	84	26
≥3	3	1	1	1	4	1
*Progression-free interval post taxane*^b^						
≤6 months	112	51	51	47	163	50
>6 months	106	49	58	53	164	50

^a^Protocol eligibility required no more than two cytotoxic regimens in the advanced setting. There was one patient in each treatment arm that did deviate from this eligibility criteria.

^b^Determined from the last taxane received.

### Activity results

**Fig. 2:**
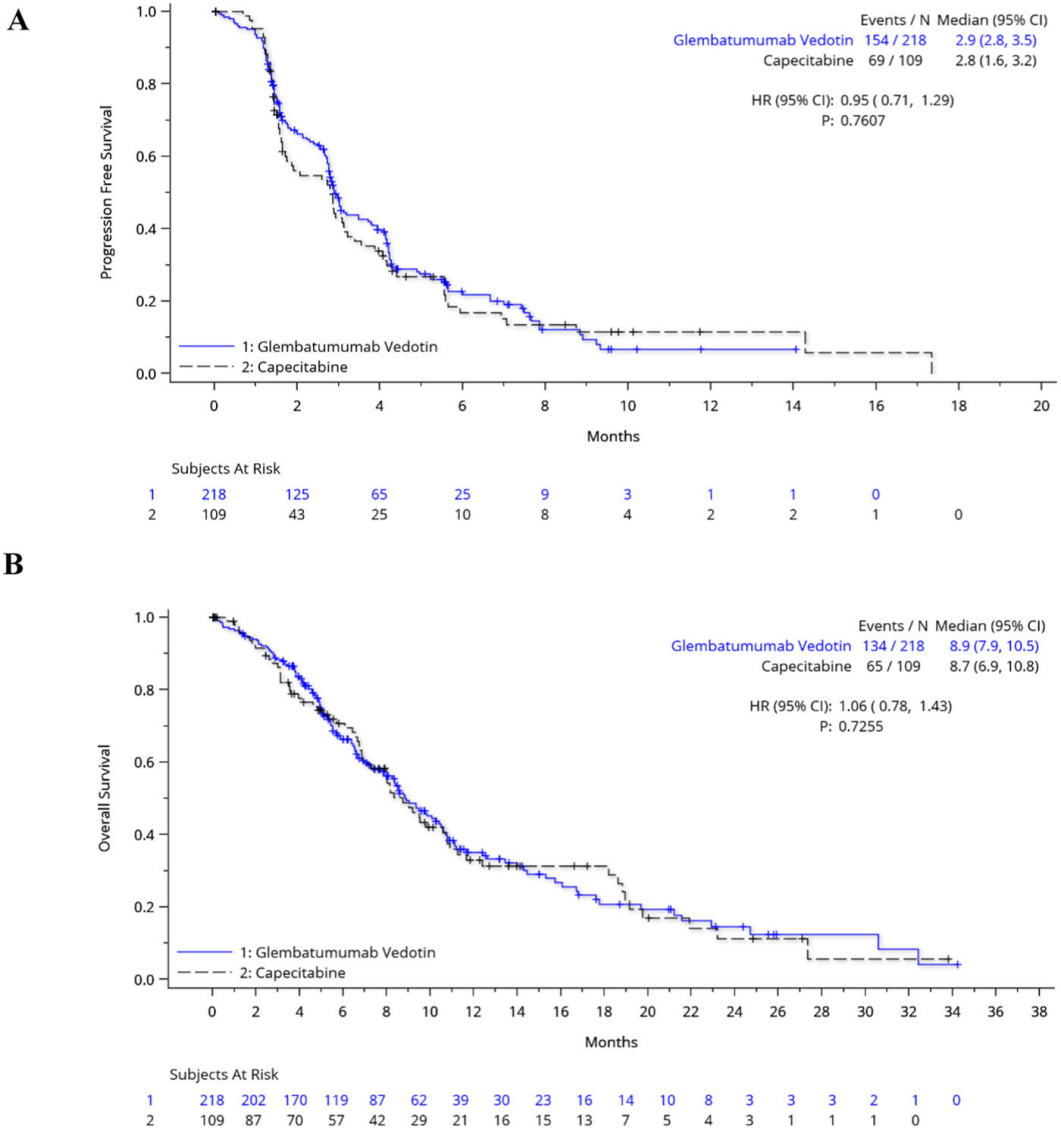
Kaplan–Meier estimates of progression-free survival and overall survival. PFS by IRC assessment (**A**) and OS (**B**) for the intention-to-treat population are shown in months. Tick marks represent censored data.

Median PFS by IRC was 2.9 months (95% CI: 2.8, 3.5) for the GV arm and 2.8 months (1.6, 3.2) months for the capecitabine arm (HR = 0.95; 95% CI: 0.71, 1.29; *p* = 0.7607) (Fig. 2A, Table 3). By investigator assessment, median PFS was 2.9 months (95% CI: 2.8, 3.1) for the GV arm and 2.7 months (95% CI: 1.8, 3.4) for the capecitabine arm (HR = 1.13; 95% CI: 0.85, 1.52; *p* = 0.4051). The secondary outcomes of OS, ORR, and duration of response (DOR) in the ITT population were similar between treatment arms.

**Table 3:** Antitumor activity.

ITT population	Glembatumumab vedotin (*n* = 218)		Capecitabine (*n* = 109)	
*PFS by IRC*				
Median, months (95% CI)	2.9	(2.8, 3.5)	2.8	(1.6, 3.2)
*OS*				
Median, months (95% CI)	8.9	(7.9, 10.5)	8.7	(6.9, 10.8)
*Duration of OS follow-up*				
Median, months (95% CI)	12.4	(10.9, 15.0)	13.6	(10.1, 20.0)
Measurable disease population	Glembatumumab vedotin (*n* = 179)		Capecitabine (*n* = 100)	
ORR by IRC, *n* (% [95% CI])	29 (16%)	11.1, 22.4	15 (15%)	8.6, 23.5
Confirmed CR, *n* (%)	1 (<1%)		3 (3%)	
Confirmed PR, *n* (%)	28 (16%)		12 (12%)	
Any response*, *n* (% [95% CI])	46 (26%)	19.5, 32.8	21 (21%)	13.5, 30.3
SD, *n* (%)	83 (46%)		27 (27%)	

Data are *n* (%).

*ITT* intention-to-treat population, includes all enrolled patients, *PFS* progression-free survival, *IRC* Independent Review Committee, *OS* overall survival, *ORR* objective response rate per RECIST 1.1, *CR* complete response, *PR* partial response, *SD* stable disease (minimum interval ≥6 weeks from baseline), *DOR* duration of response.

*Any response including those not confirmed at subsequent disease assessment.

Median OS was 8.9 (95% CI: 7.9, 10.5) months for the GV arm and 8.7 (95% CI: 6.9, 10.8) months for the capecitabine arm (HR = 1.06; 95% CI: 0.78, 1.43; *p* = 0.7255) (Fig. 2B, Table 3). Additional therapies received after study treatment and during survival follow-up were generally well balanced between study arms (Supplementary Table 1). Fifty-five percent of patients received additional anticancer medications, including 29% of patients in the GV arm who received capecitabine and ≥10% of patients in both treatment arms who received eribulin, carboplatin, or gemcitabine. The response rate to subsequent therapies was only 4%, consistent across study treatment arms.

**Fig. 3:**
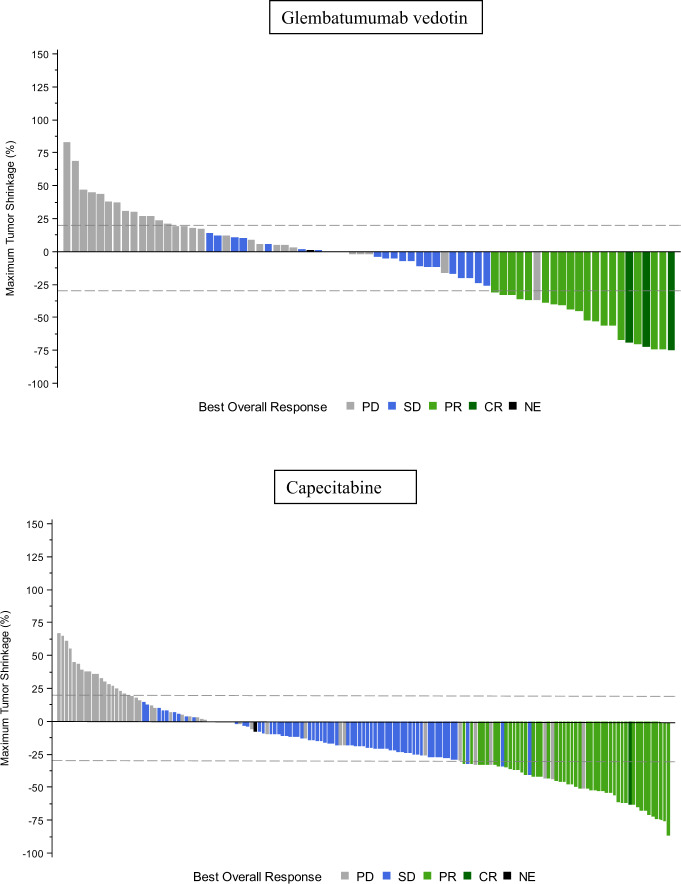
Maximum tumor shrinkage. Maximum percent tumor shrinkage is shown for patients with evaluable post-treatment measurements of RECIST 1.1 target lesions. Best overall response was determined by RECIST 1.1 criteria^33^ by Independent Review Committee. For the measurable disease population (including 15 patients in the glembatumumab arm and 11 patients in the capecitabine arm who are excluded from the waterfall plot due to lack of complete post-treatment assessment of all RECIST 1.1 target lesions), the proportion of patients with any tumor shrinkage was 113/179 (63%) in the glembatumumab vedotin arm and 39/100 (39%) in the capecitabine arm.

ORR by IRC in the measurable disease ITT population was 16% (95% CI: 11.1, 22.4) for the GV arm and 15% (95% CI: 8.6, 23.5) for the capecitabine arm (Table 3). The proportion of patients with any tumor shrinkage by IRC was 63% in the GV arm and 39% in the capecitabine arm (Fig. 3). The median DOR was 2.8 months (95% CI: 2.3, 5.5) for the GV arm and 4.2 months (95% CI: 2.7, 5.6) for the capecitabine arm. Outcomes of PFS, ORR, and DOR by investigator review are presented in Supplementary Table 2.

**Fig. 4:**
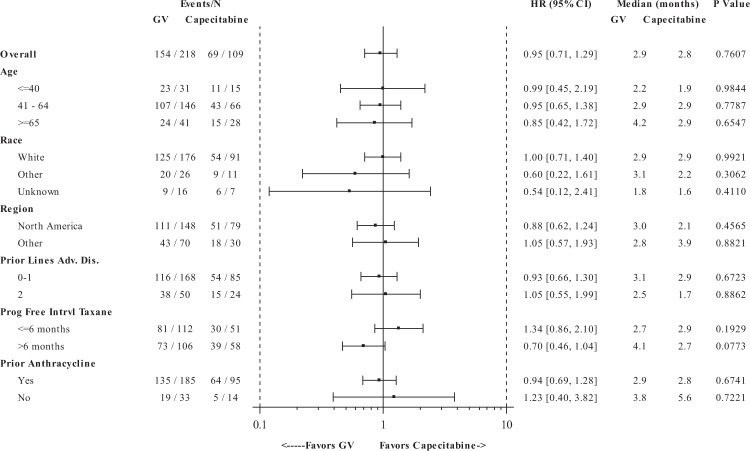
Progression-free survival by demographics and stratification factors. Progression-free survival was determined in the intention-to-treat population, based on Independent Review Committee assessments. A forest plot for progression-free survival for GV compared with capecitabine is shown by demographics and by study pre-specified randomization factors: number of prior lines of cytotoxic chemotherapy for advanced disease, progression-free interval post last receipt of taxane, and prior receipt of anthracycline. The black square represents the hazard ratio, whereas the odds ratios (95% CIs) are denoted by black lines.

**Fig. 5:**
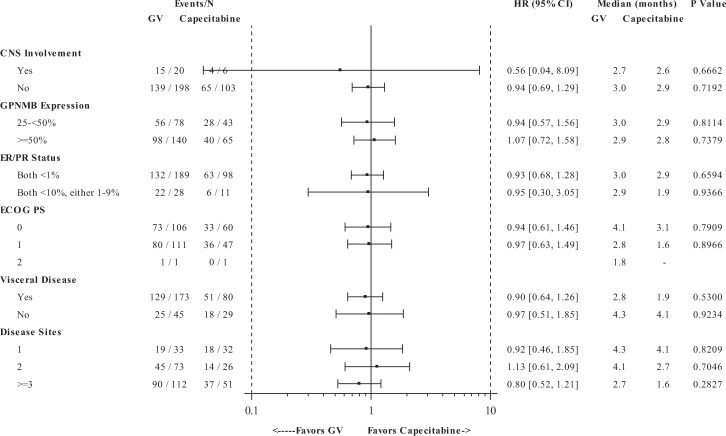
Progression-free survival by baseline disease characteristics. Progression-free survival was determined in the intention-to-treat population, based on Independent Review Committee assessments. A forest plot for progression-free survival for GV compared with capecitabine is shown by baseline disease characteristics. The black square represents the hazard ratio while the odds ratios (95% CIs) are denoted by black lines.

The pre-specified subgroup analyses did not reveal any patient subgroups with statistically significant differences in PFS and OS between treatment arms (Figs. 4, 5, Supplementary Fig. 1); however, a non-significant trend towards PFS benefit was observed for patients who did not experience progression of disease for >6 months after the last taxane therapy. Therefore, we conducted additional post hoc analyses to examine whether the extent of prior taxane exposure impacted outcome. For patients with one prior line of taxane therapy, (received in any setting and including one patient violating protocol eligibility who did not receive any taxane), the GV and capecitabine arms, respectively demonstrated median PFS of 3.0 months (95% CI: 2.8, 3.9) vs. 1.9 months (95% CI: 1.5, 3.1) (HR = 0.67; 95% CI: 0.47, 0.95) by IRC and 2.9 months (95% CI: 2.8, 3.8) vs. 1.9 months (95% CI: 1.5, 2.9) (HR = 0.79; 95% CI: 0.56, 1.11; *p* = 0.1806) by investigator review (Supplementary Fig. 2). ORR was 26% vs. 18% by IRC and 30% vs. 18% by investigator review. However, no difference in OS was observed between treatment arms in this subgroup.

Within the GV arm, early development of rash was explored to determine whether there was any association with improved outcome. For the subgroup of patients who developed rash in the first treatment cycle compared with those who did not, median PFS by IRC was 3.9 months (95% CI: 2.9, 4.2) vs. 2.8 months (95% CI: 1.6, 4.1) (HR = 0.78; 95% CI: 0.48, 1.28; *p* = 0.3277), ORR by IRC was 31% (95% CI: 21.9, 40.2) vs. 15% (95% CI: 4.4, 34.9) (*p* = 0.0475), and median OS was 13.4 months (95% CI: 10.1, 16.7) vs. 18.6 months (95% CI: 9.5, 19.7) (HR = 1.10; 5% CI: 0.64, 1.88; *p* = 0.7269). Although a significant difference in ORR was observed for this subgroup who developed rash in the first treatment cycle of GV as compared with those who developed rash to capecitabine, no differences were seen in PFS or OS for the same subgroup.

The total number of patients who had any serum samples tested for either ADC, TA, or MMAE pharmacokinetic (PK) analysis was 207. Post-infusion serum concentrations of ADC, TA, and MMAE are presented in Table 4. However, it should be noted that the MMAE concentrations are not indicative of maximum levels of exposure because MMAE levels tend to peak between 2 and 7 days post infusion^18^. No clear correlations were observed between maximum concentrations of ADC, TA, or MMAE and best overall response. Median cycle 1 TA levels appear higher in patients who experienced rash in cycle 1 as compared with those who did not (*P* = 0.005). There were no significant differences in maximum cycle 1 concentration of ADC, TA, and MMAE in subjects experiencing ≥grade 3 treatment-related toxicity vs. those who did not.

**Table 4:** Post-infusion serum concentrations.

	ADC (*N* = 12)	TA (*N* = 163)	MMAE (*N* = 48)
	Mean ± SD (range) μg/ml	Mean ± SD (range) μg/ml	Mean ± SD (range) ng/ml
Cycle 1	58.6 ± 15.5 (38.8–98.3)	49.4 ± 16.5 (21.4–127.4)	1.52 ± 1.10 (0.36–5.02)
Cycle 2	56.1 ± 11.0 (45.3–85.6)	47.3 ± 15.0 (11.8–89.9)	1.40 ± 0.83 (0.042–4.26)

*ADC* antibody–drug conjugate, *TA* total antibody, *MMAE* monomethyl auristatin E, *SD* standard deviation.

Seventy-eight (36%) patients in the GV arm and 43 (39%) patients in the capecitabine arm had tumors with 25–49% of cells expressing gpNMB, whereas 140 (64%) patients in the GV arm and 65 (60%) patients in the capecitabine arm had tumors with ≥50–100% expression. Subgroup analyses showed no difference in PFS, ORR, or OS between treatment arms based on levels of gpNMB expression.

### Safety results

Toxicity is presented in Table 5. The most common adverse events were fatigue (102 [48%]), nausea (95 [45%]), rash (95 [45%]), and alopecia (88 [41%]) in the GV arm, and diarrhea (45 [49%]), nausea (40 [44%]), palmar-plantar erythrodysesthesia (40 [44%]), and fatigue (39 [42%]) in the capecitabine arm. The most common grade 3–4 adverse events were neutropenia (59 [28%]), rash (26 [12%]), leukopenia (20 [9%]), fatigue (11 [5%]), peripheral neuropathy (10 [5%]), abdominal pain (10 [5%]), dyspnea (10 [5%]), and lymphopenia (10 [5%]) in the GV arm, and fatigue (22 [24%]), diarrhea (12 [14%]), palmar-plantar erythrodysesthesia (7 [8%]), constipation (5 [5%]), stomatitis (5 [5%]), and lymphopenia (5 [5%]) in the capecitabine arm. The most common serious adverse events were septic shock (7 [3%]), vomiting (7 [3%]), abdominal pain (6 [3%]), diarrhea (6 [3%]), dyspnea (6 [3%]), and rash, including erythematous, generalized, and maculopapular (5 [2%]), in the GV arm, and diarrhea (5 [5%]), vomiting (4 [4%]), nausea (3 [3%]), and fatigue (3 [3%]) in the capecitabine arm. Of the 199 reported deaths, the majority were owing to progressive disease (118 [54%] of 134 in the GV arm and 55 [51%] of 65 in the capecitabine arm), 22 were due to unknown/other cause (12 [6%] in the GV arm and 10 [9%] in the capecitabine arm), and 4 were due to adverse events (all GV arm). A 62-year-old patient died owing to sepsis and aspiration pneumonia considered unrelated to study treatment 20 days after the first dose of GV. Three additional patients, aged 32, 61, and 56, died owing to sepsis or septic shock, after 9 days, 12 days, and 5 months of treatment, respectively. Three of the events of fatal sepsis were preceded by treatment-related neutropenia; two were assessed as potentially related to study treatment.

**Table 5:** Toxicity.

	Glembatumumab vedotin								Capecitabine						
	(*n* = 213)								(*n* = 92)						
	Grade 1–2		Grade 3		Grade 4		Grade 5		Grade 1–2		Grade 3		Grade 4		Grade 5
Any adverse event	61	(29%)	114	(54%)	32	(15%)	4	(2%)	40	(44%)	44	(48%)	8	(9%)	0
Fatigue	91	(43%)	11	(5%)	0		0		33	(36%)	6	(7%)	0	(0%)	0
Nausea	87	(41%)	7	(3%)	0		0		37	(40%)	2	(2%)	1	(1%)	0
Rash*	69	(32%)	26	(12%)	0		0		9	(10%)	1	(1%)	0	(0%)	0
Alopecia	88	(41%)	0		0		0		1	(1%)	0		0		0
Neutropenia*	25	(12%)	38	(18%)	21	(10%)	0		4	(4%)	2	(2%)	1	(1%)	0
Pruritus*	70	(33%)	9	(4%)	0		0		4	(4%)	0		0		0
Peripheral neuropathy*	67	(32%)	10	(5%)	0		0		9	(10%)	0		1	(1%)	0
Decreased appetite	63	(30%)	1	(1%)	0		0		15	(16%)	2	(2%)	0		0
Constipation	56	(26%)	6	(3%)	0		0		13	(14%)	0	(0%)	0		0
Diarrhea	52	(24%)	7	(3%)	0		0		32	(35%)	12	(13%)	1	(1%)	0
Vomiting	44	(21%)	6	(3%)	0		0		16	(17%)	3	(3%)	1	(1%)	0
Abdominal pain*	34	(16%)	10	(5%)	0		0		19	(21%)	3	(3%)	0		0
Pyrexia*	42	(20%)	1	(1%)	0		0		12	(13%)	0		0		0
Stomatitis	30	(14%)	7	(3%)	1	(1%)	0		19	(21%)	5	(5%)	0		0
Dyspnea	26	(12%)	9	(4%)	1	(1%)	0		10	(11%)	1	(1%)	0		0
Anemia*	29	(14%)	7	(3%)	0		0		7	(8%)	3	(3%)	0		0
Leukopenia*	14	(7%)	16	(8%)	4	(2%)	0		3	(3%)	0	(0%)	0		0
Hypokalemia*	22	(10%)	6	(3%)	0		0		5	(5%)	0		4	(4%)	0
Aspartate transferase increased	16	(7%)	8	(4%)	1	(1%)	0		5	(5%)	1	(1%)	0		0
Pain	16	(8%)	7	(3%)	0		0		3	(3%)	1	(1%)	0		0
Alanine aminotransferase increased	19	(9%)	4	(2%)	0		0		1	(1%)	2	(2%)	0		0
Lymphopenia*	9	(4%)	8	(4%)	2	(1%)	0		1	(1%)	5	(5%)	0		0
Dehydration	9	(4%)	8	(4%)	1	(1%)	0		6	(7%)	1	(1%)	0		0
Blood alkaline phosphatase increased	10	(5%)	8	(4%)	0		0		2	(2%)	0		0		0
Hypophosphatemia*	8	(4%)	6	(3%)	1	(1%)	0		2	(2%)	2	(2%)	1	(1%)	0
Palmar-plantar erythrodysesthesia syndrome	9	(4%)	2	(1%)	0		0		33	(36%)	7	(8%)	0		0
Sepsis syndrome*	0		0		3	(1%)	4	(2%)	0		0		1	(1%)	0
Pulmonary embolism	0		2	(1%)	1	(1%)	0		0		4	(4%)	0		0

Data are presented for the safety population (all patients who received at least one dose of study treatment). Table shows all grade 1–2 events occurring in ≥20% of patients in either group and any grade 3–5 event occurring in ≥6 patients overall. Four patients died due to adverse events, all on the glembatumumab vedotin arm. Overall, 199 deaths were reported, 173 (87%) were owing to progressive disease (118 of the 134 reported deaths in the glembatumumab vedotin arm and 55 of the 65 reported deaths in the capecitabine arm), 22 were owing to unknown/other cause (12 in the glembatumumab vedotin arm and 10 in the capecitabine arm), and 4 due to adverse event (all on the glembatumumab vedotin arm).

*AE terms that were synonymous were combined.

Disease progression/symptomatic deterioration was the primary reason for discontinuation of GV (73%) and capecitabine (61%). Forty-one [26%] of 305 patients discontinued treatment owing to adverse events (31 [15%] of 213 in the GV arm and 10 [11%] of 92 in the capecitabine arm). Toxicity resulting in discontinuation of treatment for the GV arm vs. the capecitabine arm included peripheral neuropathy (7 [3%] vs. 0), rash (4 [2%] vs. 0), stomatitis (3 [1%] vs. 2 [2%]), sepsis (3 [1%] vs. 1 [1%]), neutropenia (1 [1%] vs. 3 [3%]), and diarrhea (0 vs. 3 [3%]). Dose reductions owing to adverse events occurred in 59 [28%] patients in the GV arm and 36 [39%] patients in the capecitabine arm. The most common events resulting in dose reductions (for the GV arm vs. the capecitabine arm) included peripheral neuropathy (17 [8%] vs. 0), palmar-plantar erythrodysesthesia (2 [1%] vs. 16 [17%]), rash (10 [5%] vs. 1 [1%]), neutropenia (7 [3%] vs. 3 [3%]), peripheral neuropathy (7 [3%]vs. 0), nausea (4 [2%] vs. 5 [5%]), fatigue (4 [2%] vs. 5 [5%]), and diarrhea (3 [1%] vs. 13 [14%]).

## Discussion

ADcs have emerged as some of the most-active therapeutics in breast cancer treatment today.^19–22^ The METRIC study is the first randomized trial evaluating the efficacy of a gpNMB-targeted therapy for metastatic gpNMB-expressing TNBC, and among the first trials evaluating a molecularly targeted therapy for TNBC. GV demonstrated more-frequent tumor shrinkage than capecitabine, however, the responses were transient and of a shorter duration than those treated with capecitabine. Furthermore, treatment with GV did not offer an advantage over capecitabine in PFS or other secondary endpoints. The safety profile of GV was similar to prior published studies, however, offered no advantage in terms of reduced toxicity over capecitabine.

The METRIC study was designed to confirm a prior exploratory analysis that suggested the greatest benefit from GV was in gpNMB-overexpressing TNBC, however, that was not confirmed in this study. Furthermore, since the design and execution of this study, 2 additional biomarker-driven studies of GV have been conducted and reported in advanced melanoma^23^, and recurrent pediatric osteosarcoma^24^, which demonstrate a lack of association of response with the intensity of gpNMB expression. Therefore, selecting patients based on the immunohistochemical expression of gpNMB is not predictive of response to GV.

Additional possibilities as to why there was no advantage to GV over capecitabine include TNBC molecular heterogeneity and drug resistance to antimicrobular agents.

TNBC is a heterogeneous disease that can be classified into distinct molecular subtypes by gene expression profiling and two of the subtypes (30% of TNBC) are characterized from a gene ontology perspective as enriched in Wnt and PI3K signaling pathways. Recently, gpNMB was found to augment WNT-1-mediated breast tumor initiation and growth by enhancing PI3K/AKT/mTOR pathway signaling and B catenin activity^25^. As not all the molecular subtypes of TNBC are enriched in those pathways it is a possibility that the target was irrelevant for a significant portion of the patients enrolled on the trial. In addition, these different subtypes have distinct responses to neoadjuvant chemotherapies with BL1 subtype exhibiting the highest pCR rate (52%) and BL2 and LAR the lowest (0 and 10%, respectively)^26^. MMAE, the payload chemotherapy for the gpNMB-expressing antibody is an anti-microtubule agent and it is unknown if cross-resistance exists with the taxanes. This raises the possibility that GV activity might be TNBC subtype specific, however, this would need to be evaluated in the future.

As study eligibility required that all patients had previously received taxane-based therapy, the treated population may have presented a certain degree of resistance to further therapy with microtubule inhibitors such as MMAE. Although there were no statistically significant findings in PFS in the predefined subgroup analyses (demographics, disease history or baseline status, and stratification factors) as shown in Figs. 4 and 5, there was a trend towards longer PFS in patients who received GV and presented with a progression-free interval >6 months vs. ≤6 months post-taxane. Consequently, an exploratory ad hoc subgroup analysis was performed suggesting the greatest benefit from GV was in the subset of patients who had only one prior taxane-containing regimen. These data support the hypothesis that GV may provide greater clinical benefit in patients who are potentially taxane-sensitive.

In addition, efflux pumps are a known mechanism of drug resistance in cancer cells, mainly through multidrug resistance protein 1 (MDR1), multidrug resistance-related protein 1 (MRP1), and breast cancer resistance protein (BCRP)^27^. MDR1 is a known efflux pump for MMAE^28^. By comparison, an ADC delivering SN-38, the active metabolite of irinotecan, was associated with a median PFS of 6.0 months in heavily pre-treated TNBC^29^. Thus, one strategy that may improve the activity of a gpNMB-targeted approach is to utilize a conjugated toxin that is less susceptible to efflux pumps or combine with agents that may overcome resistance (i.e., MDR1 inhibitors).

Other biological factors in the tumor microenvironment may also have contributed to the short duration of response to GV. Inaccessible cell surface gpNMB, insufficient intracellular MMAE accumulation, or insufficient intratumoral concentration of MMAE are possibilities. In addition, the polyclonal anti-gpNMB antibody used for the IHC screening assay may have had different binding affinities and/or specificities than the monoclonal antibody of GV.

Strategies for future research of GV could focus on a subset of patients with gpNMB-overexpressing advanced TNBC who have either previously responded to a taxane or have had minimal exposure to a taxane-containing regimen. In addition, in light of data suggesting that ADCs can cross the blood–brain barrier owing to disruption of the vasculature by malignant processes^14,30,31^, and the possible role of gpNMB in the development of brain metastases^32^, there may be an opportunity for GV to address the large unmet need for patients with breast cancer brain metastases.

## Methods

### Study design

METRIC was a phase 2b, open-label, randomized study conducted at 120 sites in the United States, Canada, Australia, United Kingdom, France, Spain, Belgium, Germany, and Italy. The study was conducted at each of the participating institutions according to the Declaration of Helsinki and Good Clinical Practice Guidelines, after approval by local institutional/ethics review boards. The full trial protocol can be found in the Appendix.

### Participants

Eligible patients were ≥18 years of age with metastatic TNBC that overexpressed gpNMB, defined as ≥25% tumor epithelial cells staining positive in a tumor sample obtained in the advanced disease setting, (advanced disease was intended to include locally advanced, metastatic, or recurrent disease). Triple-negative status was confirmed in the advanced setting and defined by ER and PR expression in <10% cells by IHC and negative HER2 status defined as an IHC score of 0 or 1+, or in situ hybridization (ISH) copy number <4.0 signals/cell, or ISH HER2/CEP17^b^ ratio <2.0 with average copy number <4.0 signals/cell. Patients with low ER and PR expression, i.e., 1–9%, must have been deemed appropriate candidates for chemotherapy by the investigator.

Patients must have received a prior taxane and, unless not clinically indicated, anthracycline, with ≤2 lines of prior chemotherapy in the advanced setting. Documented disease progression must have occurred during or subsequent to the last anticancer regimen received. Patients were required to have measurable disease for assessment by RECIST 1.1 criteria^33^. Patients were excluded from the study if they had progression/recurrence of breast cancer within 3 months of completion of neoadjuvant or adjuvant chemotherapy; had known brain metastases unless previously treated and asymptomatic for ≥2 months; or persistent neuropathy >grade 1 at time of randomization.

All patients signed written informed consent prior to any protocol-specific procedures.

### Randomization and masking

Patients were stratified by a number of prior lines of chemotherapy for advanced disease (0–1 vs. 2), by prior receipt of anthracycline (yes vs. no), and by the progression-free interval after last taxane received (≤6 vs. >6 months). Patients were randomly assigned (2:1) to the treatment groups using a pre-specified randomization sequence, block size of 6, via interactive response technology. As this was open-label, there was no masking of trial participants or investigators. The IRC and biostatistical team at the study sponsor were masked to treatment assignments until after study closure.

### Procedures

GV (Celldex Therapeutics, Inc., Fall River, MA) was administered every 3 weeks as a 90-minute intravenous infusion, starting dose of 1.88 mg/kg. Dose reductions to 1.3 and 1.0 mg/kg were allowed for toxicity. Capecitabine (Roche, Belgium and Accord, UK and India) was supplied by Celldex to most clinical sites in the EU, whereas the remaining institutions utilized available commercial supplies. Capecitabine was administered per package insert recommendations, starting dose of 1250 mg/m^2^ orally twice daily for 2 weeks followed by a 1-week rest period. Subsequent capecitabine treatment was dictated by tolerance and institutional practice. Study treatment continued until disease progression or intolerance.

Safety assessments included physical examination, vital signs, hematology, and blood chemistry at ~3-week intervals throughout treatment; urinalysis ~6 weeks; and electrocardiogram at baseline and end of treatment. Adverse events were graded according to NCI-CTCAE version 4.0.

Radiographic assessments were performed within 4 weeks prior to treatment, every 6 weeks for 6 months, and every 9 weeks thereafter, or until progression of disease. Tumor response and progression were assessed according to RECIST 1.1 criteria. Local investigator assessments guided treatment decisions, whereas study analyses were based on retrospective IRC (Bioclinica, Princeton, NJ) assessments masked to treatment assignments. Patients who experienced disease progression were followed for survival until study closure.

Serum samples for PK analysis were drawn for patients who received GV before and after every infusion. Intact ADC and total antibody (TA) were quantified using enzyme-linked immunosorbent assays. Free MMAE was quantified using liquid chromatography/mass spectrometry. The assay sensitivities were 0.32 μg/mL, 0.50 μg/mL, and 0.05 ng/ml for ADC, TA, and free MMAE, respectively. Peripheral blood mononuclear cell samples were collected from a subset of centers in the US, prior to GV dosing. The analysis included the examination of GPNMB expression on myeloid suppressor cells.

Prospective analysis of gpNMB expression was performed by IHC at a central laboratory (Neogenomics, Aliso Viejo, CA) using tumor tissue from the advanced disease setting, either archived or obtained at baseline. Samples on 3–5 micron slides were deparaffinized, rehydrated, and pre-treated using Envision FLEX target retrieval solution (Agilent, Santa Clara, CA). After proper washing, slides were incubated with the goat anti-human gpNMB antibody (R&D Systems, Minneapolis, MN). Visualization was achieved using horseradish peroxidase-labeled rabbit anti-goat (Invitrogen, Carlsbad, CA) and DAB+ chromogen (Agilent). A duplicate slide from each sample was processed similarly but replaced the goat anti-gpNMB antibody with normal goat IgG (R&D Systems) to serve as an isotypic control. After slides were counterstained with hematoxylin, gpNMB staining was scored by two pathologists, recording the percentage of malignant epithelial cells staining positive. The disparity in results between the two pathologists influencing a patient’s eligibility status was adjudicated by the two pathologists under a multi-head microscope or by a third pathologist.

### Outcomes

The primary study objective was to demonstrate that GV improves PFS (defined as the time from the date of randomization to documented disease progression, or death owing to any cause, whichever is earlier) as compared with capecitabine. PFS events were determined retrospectively in a masked fashion by the central IRC according to RECIST 1.1 criteria. Secondary study objectives included the effect of GV as compared with capecitabine on objective response rate (ORR; the proportion of patients among those with measurable disease at baseline achieving a confirmed complete response (CR) or partial response (PR) per RECIST 1.1 criteria), DOR (the number of months from the start date of PR or CR [whichever is recorded first] to the first date that PD or death is documented]), and overall survival, as well as to further characterize the safety, and obtain PK parameters and explore the relationships between exposure and safety and activity parameters. Activity analyses were performed according to IRC assessments.

### Statistical considerations

For the purpose of the study’s sample size calculation, it was hypothesized that median PFS would be 4.0 months for patients with metastatic, gpNMB-expressing TNBC treated with capecitabine alone, and GV would increase median PFS in such patients by 2.25 months (i.e., from 4.0 to 6.25 months). Thus, 203 PFS events (total of two arms) were calculated to provide 85% power to detect a hypothesized hazard ratio of 0.64 with two-sided type I error 0.05.

The primary analysis of PFS was performed for the intention-to-treat population, which consisted of all randomly assigned patients. Patients who initiated alternate anticancer therapy in absence of documented progression were censored at the latest disease assessment prior to initiation of such therapy. Patients who were last known to be alive and progression-free were censored at the latest disease assessment. PFS was summarized using the Kaplan–Meier method and presented by the treatment group. The primary inferential comparison of this endpoint used the stratified log-rank test stratified by the actual stratification factors corrected in the clinical database after randomization. Estimation of the hazard ratio for treatment and its corresponding 95% confidence interval (CI) was determined using a stratified Cox proportional hazards model with the same stratification factors (SAS version 9.4).

The secondary endpoints of ORR and DOR were based on the measurable disease population, which included patients with at least one measurable lesion on baseline evaluation. Exact 95% CI of ORR was calculated by treatment arm using the Clopper–Pearson method. Inferential comparisons of the observed ORRs were made using the Cochran–Mantel Haenszel chi-square test, stratified by the randomization stratification factors. DOR was estimated using the Kaplan–Meier method.

To determine whether there was any association between maximum serum concentrations and clinical activity or toxicity of GV, analyses were performed with peak serum concentrations during cycle 1 for each analyte vs. best overall response per RECIST 1.1 (Kruskal–Wallis, one-way analysis of variance) or ≥grade 3 CTCAE treatment-related adverse events occurring in cycle 1 (Mann–Whitney test).

Subgroup analyses were planned according to baseline characteristics including the randomization stratification factors, demographics, gpNMB and ER/PR expression levels, extent of disease, performance status, and geographical region. As early development of rash was associated with improved outcome in prior studies of GV^16,17^, an additional pre-planned subgroup analysis examined outcomes dependent on the development of treatment-related rash during the first treatment cycle. A post hoc exploratory analysis investigating outcomes by the number of lines of prior taxane-containing regimens was also performed.

Safety analyses included all patients who received at least one dose of study treatment. An independent data monitoring committee comprised of two physicians and one biostatistician reviewed cumulative safety data approximately every 6 months throughout study conduct. SAS version 9.4 was used for all statistical analyses. The trial is registered with ClinicalTrials.gov (NCT01997333).

### Reporting summary

Further information on experimental design is available in the Nature Research Reporting Summary linked to this paper.

## Supplementary information

Supplementary Information

Reporting Summary Checklist

## Data Availability

The data generated and analyzed during this study are described in the following data record: 10.6084/m9.figshare.13154069^34^. The data are available from the company which funded the study—Celldex Therapeutics, Inc (https://www.celldex.com/)—on reasonable request for a period of 3 years following publication. After this period Celldex does not commit to making the data available as the compound has been discontinued from development. For all data requests please contact Celldex at: info@celldex.com.

## Code availability

The study protocol and statistical analysis plan are available at: https://clinicaltrials.gov/ct2/show/NCT01997333.
