# Supplementary material for: Glembatumumab vedotin for patients with metastatic, gpNMB overexpressing, triple-negative breast cancer (“METRIC”): a randomized multicenter study
Source: NPJ Breast Cancer. 2021 May 20;7:57. doi: 10.1038/s41523-021-00244-6 (PMC8137923; doi:10.1038/s41523-021-00244-6)
Supplement: Supplementary file 1 — Supplementary Information [file 41523_2021_244_MOESM1_ESM.pdf]

	Events/N GV	Events/N Capecitabine	HR (95% CI)	Median (months) GV	Median (months) Capecitabine	P Value
**Overall**	134 / 218	65 / 109	1.06 [0.78, 1.43]	8.9	8.7	0.7255
**Age**						
<=40	23 / 31	12 / 15	1.95 [0.86, 4.43]	5.5	8.6	0.1037
41 - 64	87 / 146	36 / 66	0.85 [0.57, 1.27]	9.0	8.2	0.4255
>=65	24 / 41	17 / 28	1.53 [0.76, 3.07]	10.7	10.8	0.2270
**Race**						
White	111 / 176	51 / 91	1.12 [0.80, 1.58]	8.8	9.0	0.5095
Other	15 / 26	11 / 11	0.76 [0.29, 2.02]	9.9	8.2	0.5809
Unknown	8 / 16	3 / 7	1.07 [0.23, 4.98]	6.5	10.8	0.9325
**Region**						
North America	100 / 148	53 / 79	1.00 [0.71, 1.42]	8.8	8.0	0.9958
Other	34 / 70	12 / 30	1.60 [0.75, 3.40]	10.6	11.7	0.2218
**Prior Lines Adv. Dis.**						
0-1	102 / 168	48 / 85	1.11 [0.78, 1.58]	9.8	9.5	0.5650
2	32 / 50	17 / 24	0.91 [0.50, 1.67]	7.1	6.7	0.7614
**Prog Free Intrvl Taxane**						
<=6 months	74 / 112	32 / 51	1.24 [0.80, 1.91]	6.9	8.3	0.3345
>6 months	60 / 106	33 / 58	0.90 [0.58, 1.38]	10.3	9.2	0.6173
**Prior Anthracycline**						
Yes	121 / 185	58 / 95	1.15 [0.84, 1.59]	8.4	8.7	0.3839
No	13 / 33	7 / 14	0.41 [0.15, 1.08]	16.8	8.0	0.0628

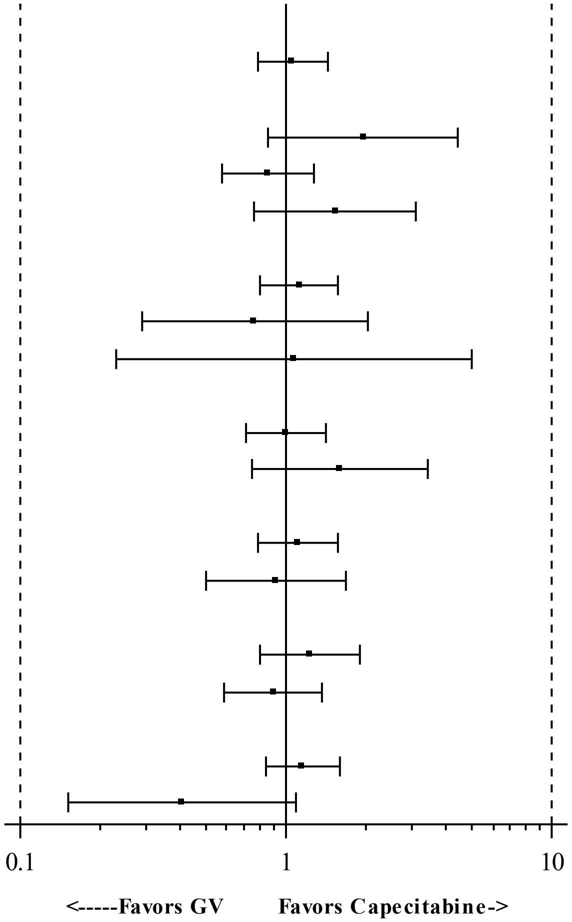

	Events/N GV	Events/N Capecitabine	HR (95% CI)	Median (months) GV	Median (months) Capecitabine	P Value
**CNS Involvement**						
Yes	15 / 20	4 / 6	1.56 [0.21, 11.70]	5.5	5.7	0.6622
No	119 / 198	61 / 103	1.03 [0.74, 1.42]	9.4	8.7	0.8713
**GPNMB Expression**						
25-<50%	49 / 78	25 / 43	1.16 [0.69, 1.95]	8.6	8.1	0.5748
>=50%	85 / 140	39 / 65	1.25 [0.83, 1.87]	9.0	8.3	0.2816
**ER/PR Status**						
Both <1%	116 / 189	60 / 98	1.06 [0.77, 1.46]	8.8	8.7	0.7332
Both <10%, either 1-9%	17 / 28	5 / 11	1.36 [0.37, 5.06]	10.3	6.7	0.6452
**ECOG PS**						
0	59 / 106	33 / 60	1.08 [0.68, 1.69]	10.5	10.6	0.7485
1	74 / 111	32 / 47	1.04 [0.67, 1.62]	7.3	7.1	0.8609
2	1 / 1	0 / 1		4.2	-	
**Visceral Disease**						
Yes	115 / 173	50 / 80	1.01 [0.72, 1.43]	8.4	8.0	0.9354
No	19 / 45	15 / 29	0.84 [0.39, 1.80]	14.3	11.7	0.6568
**Disease Sites**						
1	15 / 33	14 / 32	1.09 [0.49, 2.41]	13.4	18.6	0.8307
2	35 / 73	14 / 26	0.97 [0.50, 1.88]	11.1	9.5	0.9223
>=3	84 / 112	37 / 51	0.93 [0.62, 1.39]	7.0	6.8	0.7179

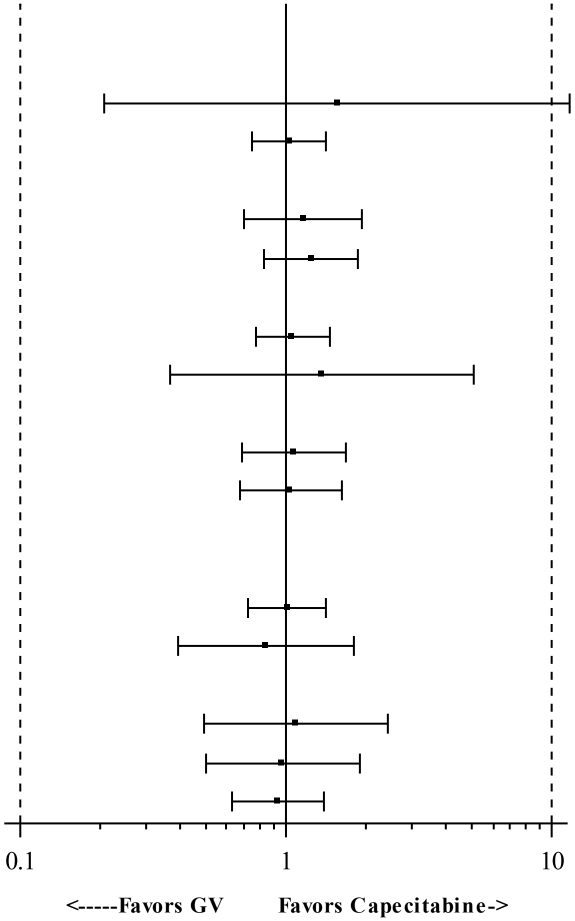

Supplementary Figure 1. Overall Survival by Subgroup Analysis

Overall survival was determined in the Intention-to-Treat Population. Subgroup analysis was performed by demographics and by study prespecified randomization factors: number of prior lines of cytotoxic chemotherapy for advanced disease, progression-free interval post last receipt of taxane, and prior receipt of anthracycline.

A

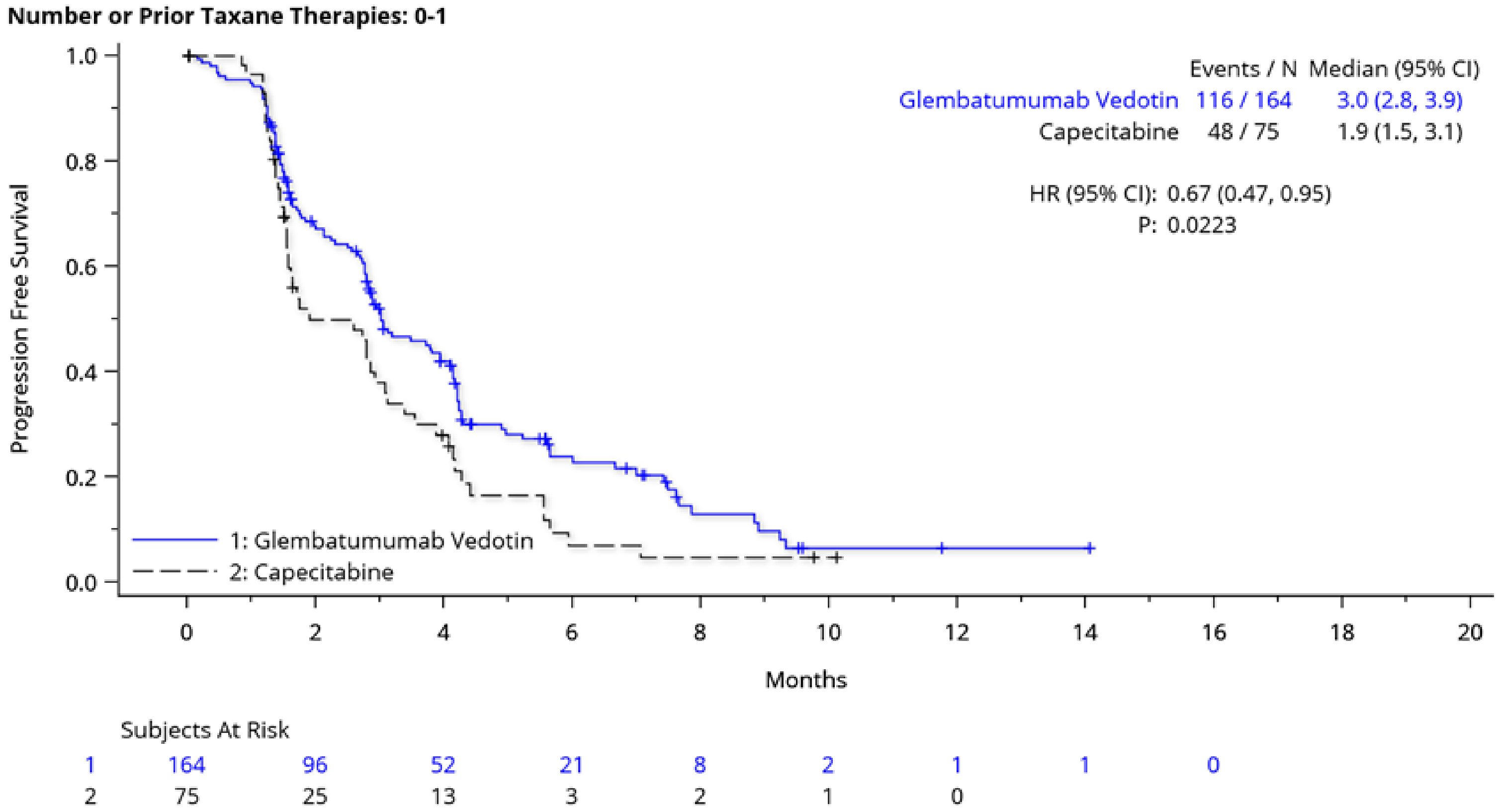
Number or Prior Taxane Therapies: 0-1
Events / N Median (95% CI)
Glembatumumab Vedotin 116 / 164 3.0 (2.8, 3.9)
Capecitabine 48 / 75 1.9 (1.5, 3.1)
HR (95% CI): 0.67 (0.47, 0.95)
P: 0.0223
1: Glembatumumab Vedotin
2: Capecitabine
Progression Free Survival
1.0
0.8
0.6
0.4
0.2
0.0
0
2
4
6
8
10
12
14
16
18
20
Months
Subjects At Risk
1 164 96 52 21 8 2 1 1 0
2 75 25 13 3 2 1 0

B

Number or Prior Taxane Therapies: >=2

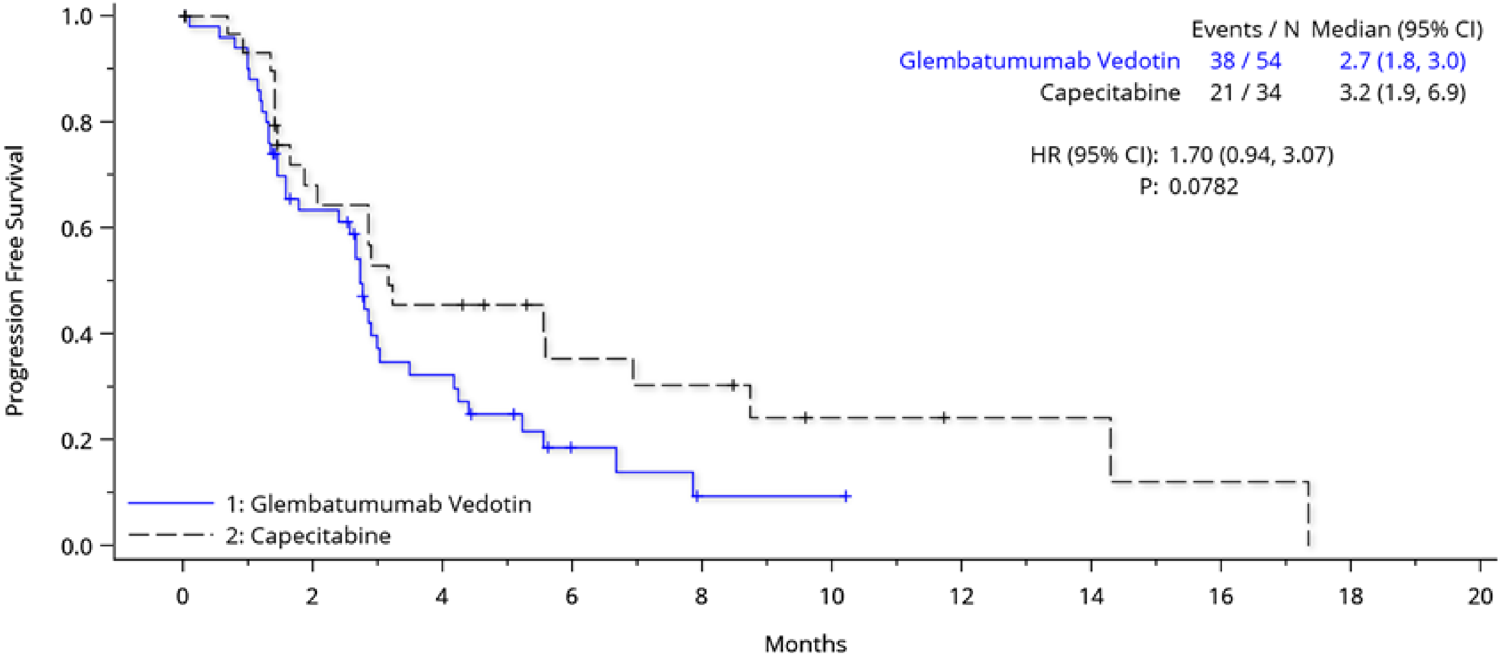

Subjects At Risk

	0	2	4	6	8	10	12	14	16	18
1	54	29	13	4	1	1	0			
2	34	18	12	7	6	3	2	2	1	0

C

Number or Prior Taxane Therapies: 0-1

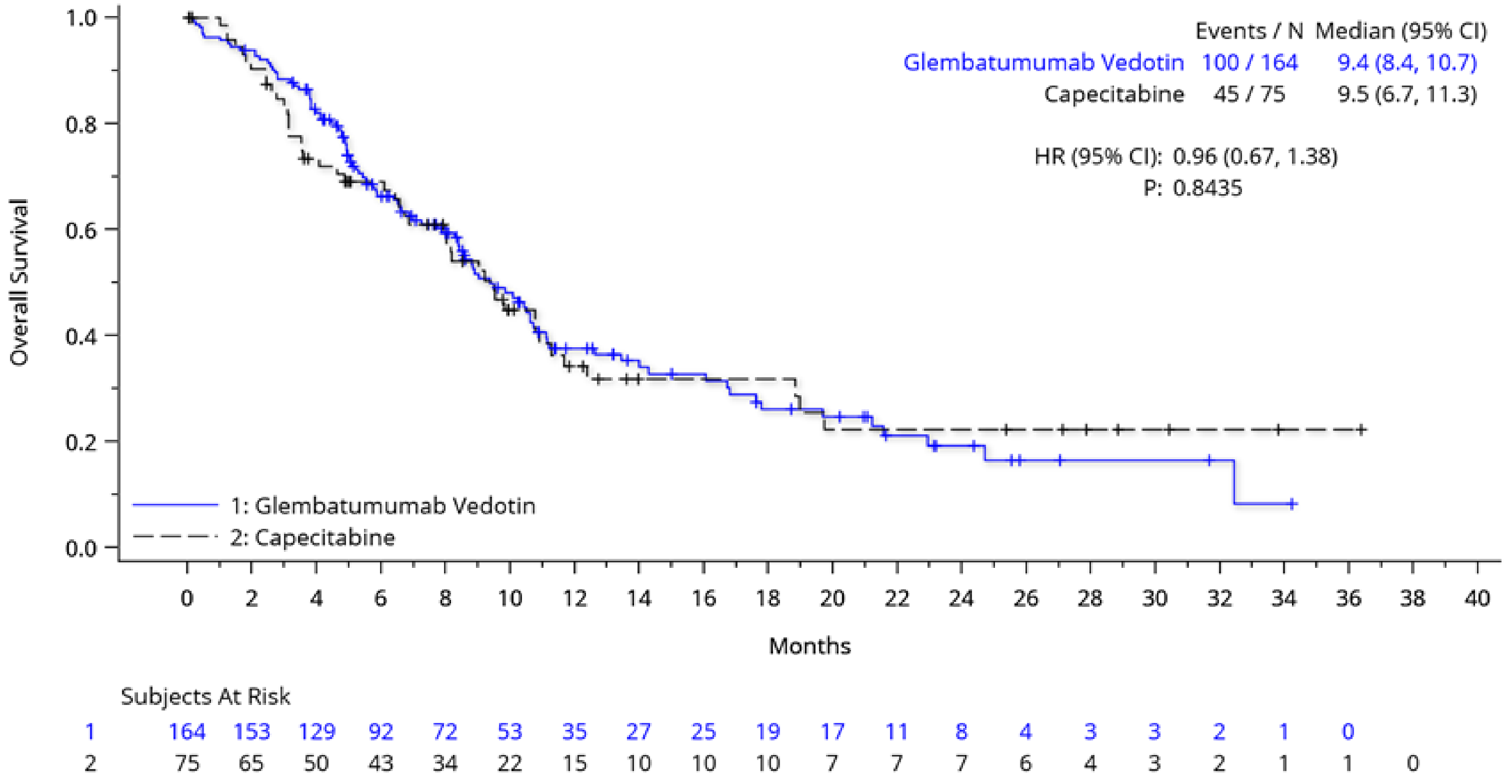

D

Number or Prior Taxane Therapies: >=2

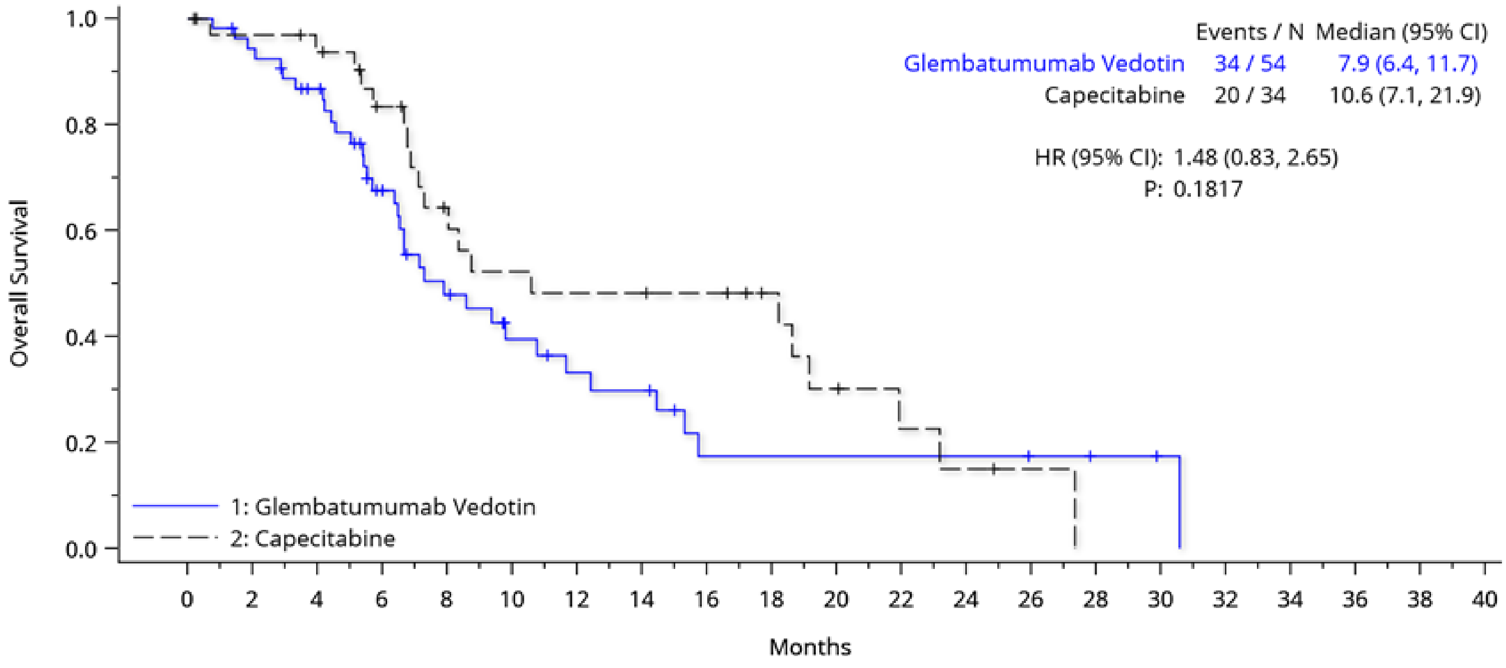

Subjects At Risk

	0	2	4	6	8	10	12	14	16	18	20	22	24	26	28	30	32
1	54	50	43	29	19	13	10	9	4	4	4	4	4	3	2	1	0
2	34	31	29	23	16	13	12	12	11	8	5	3	2	1	0		

Supplementary Figure 2. Kaplan-Meier Estimates of Progression-Free and Overall Survival by Prior Lines of Taxane

Exploratory post-hoc analysis of progression-free survival and overall survival. PFS by IRC assessment by subgroups (A) 0-1 prior line of a taxane-containing regimen and (B) two prior lines of a taxane-containing regimen. OS by subgroups (C) 0-1 prior line of a taxane-containing regimen and (D) two prior lines of a taxane-containing regimen. Tick marks represent censored data.

Supplementary Table 1. Additional Anticancer Medications Received in the Post-Treatment Follow-Up Period

	Glembatumumab vedotin	Capecitabine	Overall
ITT Population	N=218	N=109	N=327
Capecitabine	29%	3%	20%
Eribulin[1]	18%	25%	21%
Gemcitabine[2]	14%	10%	13%
Carboplatin	13%	13%	13%
Doxorubicin[3]	9%	8%	9%
Paclitaxel[4]	7%	3%	5%
Cyclophosphamide	4%	5%	4%
Pembrolizumab	4%	4%	4%
Vinorelbine[5]	4%	5%	4%
Investigational	3%	3%	3%
Bevacizumab	3%	1%	2%
Docetaxel	2%	1%	2%
Ixabepilone	1%	4%	2%
Olaparib	1%	4%	2%
Everolimus	1%	2%	1%
Fluorouracil	1%	2%	1%
Methotrexate	1%	2%	1%
Cisplatin	1%	1%	1%
Epirubicin	1%	1%	1%
Irinotecan	1%	1%	1%
Monoclonal antibodies (investigational)	1%	1%	1%
Nivolumab	1%	1%	1%
Pertuzumab	1%	1%	1%
Pexidartinib	1%	1%	1%
Sacituzumab govitecan	1%	1%	1%
Trastuzumab	1%	1%	1%
Etoposide	1%	0%	<1%
Pemetrexed	1%	0%	<1%
Seviteronel	1%	0%	<1%
Ibrutinib	0%	1%	<1%
Mitomycin	0%	1%	<1%

1. Includes eribulin mesylate
2. Includes gemcitabine hydrochloride
3. Includes doxorubicin hydrochloride and pegylated liposomal doxorubicin hydrochloride
4. Includes paclitaxel albumin
5. Includes vinorelbine tartrate

Supplementary Table 2. Antitumor Activity by Investigator Review

	Glembatumumab vedotin		Capecitabine	
ITT Population	N=218		N=109	
PFS				
Median, months (95% CI)	2.9	(2.8, 3.1)	2.7	(1.8, 3.4)
Measurable Disease ITT Population	N=214		N=108	
ORR, n (% [95% CI])	63 (29%)	23.4, 36.0	23 (21%)	14.0, 30.2
Confirmed CR, n (%)	5 (2%)		4 (4%)	
Confirmed PR, n (%)	58 (27%)		19 (18%)	
Any Response*, n (% [95% CI])	63 (29%)	23.4, 36.0	23 (21%)	14.0, 30.2
SD, n (%)	73 (34%)		28 (26%)	

Abbreviations: ITT, Intention-to-Treat population, includes all enrolled patients; PFS, Progression-Free Survival; ORR, Objective Response Rate per RECIST 1.1; CR, Complete Response; PR, Partial Response; SD, Stable Disease (minimum interval ≥ 6 weeks from baseline); DOR, Duration of Response.
*Any response including those not confirmed at subsequent disease assessment
