# Supplementary material for: Glembatumumab vedotin for patients with metastatic, gpNMB overexpressing, triple-negative breast cancer (“METRIC”): a randomized multicenter study
Source: NPJ Breast Cancer. 2021 May 20;7:57. doi: 10.1038/s41523-021-00244-6 (PMC8137923; doi:10.1038/s41523-021-00244-6)
Supplement: Supplementary file 2 — Reporting Summary Checklist [file 41523_2021_244_MOESM2_ESM.pdf]

nature research

Corresponding author(s): Linda T. Vahdat, MD., MBA

Last updated by author(s): Nov 11, 2020

Reporting Summary

Nature Research wishes to improve the reproducibility of the work that we publish. This form provides structure for consistency and transparency in reporting. For further information on Nature Research policies, see Authors & Referees and the Editorial Policy Checklist.

Statistics

For all statistical analyses, confirm that the following items are present in the figure legend, table legend, main text, or Methods section.

n/a	Confirmed	
☐	☒	The exact sample size (*n*) for each experimental group/condition, given as a discrete number and unit of measurement
☒	☐	A statement on whether measurements were taken from distinct samples or whether the same sample was measured repeatedly
☐	☒	The statistical test(s) used AND whether they are one- or two-sided<br>*Only common tests should be described solely by name; describe more complex techniques in the Methods section.*
☐	☒	A description of all covariates tested
☐	☒	A description of any assumptions or corrections, such as tests of normality and adjustment for multiple comparisons
☐	☒	A full description of the statistical parameters including central tendency (e.g. means) or other basic estimates (e.g. regression coefficient) AND variation (e.g. standard deviation) or associated estimates of uncertainty (e.g. confidence intervals)
☐	☒	For null hypothesis testing, the test statistic (e.g. *F*, *t*, *r*) with confidence intervals, effect sizes, degrees of freedom and *P* value noted<br>*Give P values as exact values whenever suitable.*
☒	☐	For Bayesian analysis, information on the choice of priors and Markov chain Monte Carlo settings
☒	☐	For hierarchical and complex designs, identification of the appropriate level for tests and full reporting of outcomes
☐	☒	Estimates of effect sizes (e.g. Cohen's *d*, Pearson's *r*), indicating how they were calculated

Our web collection on statistics for biologists contains articles on many of the points above.

Software and code

Policy information about availability of computer code

Data collection	Metidata Rave
Data analysis	SAS V. 9.4

For manuscripts utilizing custom algorithms or software that are central to the research but not yet described in published literature, software must be made available to editors/reviewers. We strongly encourage code deposition in a community repository (e.g. GitHub). See the Nature Research guidelines for submitting code & software for further information.

Data

Policy information about availability of data

All manuscripts must include a data availability statement. This statement should provide the following information, where applicable:

- Accession codes, unique identifiers, or web links for publicly available datasets
- A list of figures that have associated raw data
- A description of any restrictions on data availability

The data generated and analysed during this study are described in the following data record: https://doi.org/10.6084/m9.figshare.1315406934. The data are available from the company which funded the study--Celldex Therapeutics, Inc (https://www.celldex.com/)--on reasonable request for a period of 3 years following publication. After this period Celldex does not commit to making the data available as the compound has been discontinued from development. For all data requests please contact Celldex at: info@celldex.com.

Field-specific reporting

Please select the one below that is the best fit for your research. If you are not sure, read the appropriate sections before making your selection.

☒ Life sciences ☐ Behavioural & social sciences ☐ Ecological, evolutionary & environmental sciences

For a reference copy of the document with all sections, see nature.com/documents/nr-reporting-summary-flat.pdf

Life sciences study design

All studies must disclose on these points even when the disclosure is negative.

Sample size	For the purpose of the study's sample size calculation, it was hypothesized that median PFS would be 4.0 months for patients with metastatic, gpNMB-expressing TNBC treated with capecitabine alone, and glembatumumab vedotin would increase median PFS in such patients by 2.25 months (i.e., from 4.0 to 6.25 months). Thus, 203 PFS events (total of two arms) was calculated to provide 85% power to detect a hypothesized hazard ratio of 0.64 with 2-sided type I error 0.05.
Data exclusions	No data were excluded
Replication	IRC Chart; metastatic GPNMB-expressing triple-negative breast cancer;tumor assessments be performed at six week intervals for six months and nine week intervals thereafter
Randomization	Patients were randomized 2:1 to glembatumumab vedotin or control
Blinding	Not blinded- primary analysis based on independent data review of 56 patient scans

Reporting for specific materials, systems and methods

We require information from authors about some types of materials, experimental systems and methods used in many studies. Here, indicate whether each material, system or method listed is relevant to your study. If you are not sure if a list item applies to your research, read the appropriate section before selecting a response.

Materials & experimental systems

n/a	Involved in the study	
☒	☐	Antibodies
☒	☐	Eukaryotic cell lines
☒	☐	Palaeontology
☒	☐	Animals and other organisms
☐	☒	Human research participants
☐	☒	Clinical data

Methods

n/a	Involved in the study	
☒	☐	ChIP-seq
☒	☐	Flow cytometry
☒	☐	MRI-based neuroimaging

Human research participants

Policy information about studies involving human research participants

Population characteristics	Eligible patients were ≥ 18 years of age with progressive, metastatic Triple Negative Breast Cancer (TNBC) that overexpressed gpNMB who had received no more than 2 lines of prior chemotherapy in the advanced disease setting. Patients must have received a prior taxane and anthracycline, unless not clinically indicated.
Recruitment	Prospective patients were identified by the participating investigators and screened for study entry following informed consent.
Ethics oversight	The protocol was reviewed and approved by relevant regulatory bodies and independent ethics committees/institutional review boards prior to initiation.

Note that full information on the approval of the study protocol must also be provided in the manuscript.

Clinical data

Policy information about clinical studies

All manuscripts should comply with the ICMJE guidelines for publication of clinical research and a completed CONSORT checklist must be included with all submissions.

Clinical trial registration	NCT01997333
Study protocol	On clinicaltrials.gov

Data collection

Investigator staff were provided access to an electronic Case Report Form (CRF) within Medidata Rave for the recording and collection of data. An electronic audit trail exists within the system to record all data entries and changes. All entries in the CRF were verified against source documents. The investigator signed the CRFs to indicate that they were complete and accurate. Additionally, a portion of the investigative sites were subject to independent audits to confirm compliance with regulations and GCP guidelines.

Data collection for each patient initiated with patient screening and continued through death, study closure of patient discontinuation. Patients were randomized between February 2014 and August 2017. Patient treatment and follow up continued through August 2018. The data cut for the primary analysis occurred in November 2017 when it was determined that the pre-specified number of events had been reached for the primary analysis. The primary analysis was conducted in April 2018.

Outcomes

The primary and secondary outcome measures were selected based on clinical relevant in the population under study and defined in the study protocol. The protocol was reviewed and approved by relevant regulatory bodies and ethics committees prior to initiation. The methods of assessment are defined in the protocol and statistical analysis plan which are available on clinicaltrials.gov (NCT01997333).
